# Multi-hit *TP53* confers the poorest survival in multiple myeloma in the era of novel therapies

**DOI:** 10.1186/s10020-025-01392-2

**Published:** 2025-11-29

**Authors:** Romana Nesnadna, Anna Petrackova, Jiri Minarik, Vojtech Latal, Jirina Manakova, Tomas Papajik, Eva Kriegova

**Affiliations:** 1 Department of Immunology, Faculty of Medicine and Dentistry, Palacky University Olomouc and University Hospital Olomouc, Olomouc, Czech Republic; 2 Department of Hemato-Oncology, Faculty of Medicine and Dentistry, Palacky University and University Hospital Olomouc, Olomouc, Czech Republic

**Keywords:** Multi-hit *TP53*, Multiple Myeloma, High-risk Myeloma, Next-Generation Sequencing

## Abstract

Multiple myeloma (MM) with high-risk (HR) genetic abnormalities has poor prognosis, despite the use of novel therapeutic agents. However, the individual contribution of specific HR genetic abnormalities or their co-occurrence to poor outcomes, especially in the era of novel agents, remains unclear. This study evaluated the impact of multi-hit *TP53* (del(17p) and *TP53* mutation or ≥ 2 *TP53* mutations) compared with other HR abnormalities on progression-free survival (PFS), overall survival (OS) and blood signature in a real-world cohort of 204 patients with MM treated with novel agents (median follow-up 28 months). Patients with multi-hit *TP53* (10.4%) had the shortest PFS and OS compared with those with single HR abnormalities (*p* ≤ 0.011) or with co-occurrence of ≥ 2 other HR abnormalities (*p* ≤ 0.002), regardless of therapy line. The relative risk of early progression in patients with multi-hit *TP53* was almost three times higher than that of patients with other HR abnormalities. The prevalence of *TP53* alterations increased in later disease stages: multi-hit *TP53* was detected in 7.6% of patients with ≤ 1 prior therapy line and in 36.4% of patients with ≥ 2 prior lines. Patients with multi-hit *TP53* also differed in blood signature, particularly in counts of white blood cells, lymphocytes, serum creatinine and β2-microglobulin levels compared with those with other HR abnormalities. In conclusion, multi-hit *TP53* is associated with the poorest survival among all HR subgroups in MM. Considering that *TP53* alterations accumulate during MM progression and are associated with drug resistance even in the context of novel therapies, our study further emphasizes the need for routine evaluation of both del(17p) and *TP53* mutations. Patients with multi-hit *TP53* should be prioritized for inclusion in trials of novel therapeutic strategies.

**Supplementary Information:**

The online version contains supplementary material available at 10.1186/s10020-025-01392-2.

## Introduction

Multiple myeloma (MM) is the second most frequent haematological malignancy in adults in Western countries (Donk et al. 2021). The prognosis of patients with MM has improved substantially because of the novel drug agents approved in the last 20 years. Nevertheless, patients with high-risk (HR) disease continue to have poor survival and represent an unmet clinical need (Corre et al. 2021).

Recent studies have defined HR disease as the presence of any of the following genetic abnormalities: t(4;14), t(14;16), t(14;20), del(1p), del(17p) or *TP53* mutation (*TP53*mut) or combination of gain/amplification (amp) 1q with other HR abnormalities (Rajkumar 2024; Rees et al. 2024; Avet-Loiseau et al. 2025). Moreover, the co-occurrence of any two (double-hit), three or more (triple-hit) HR abnormalities has been termed ultra-HR MM (Rajkumar 2024), as these patients have worse clinical outcomes than those with one HR abnormality (Kaiser et al. 2025; Rajkumar 2020). However, it remains unclear to what extent different HR abnormalities, or their co-occurrence, contribute to treatment resistance and disease progression.

Among HR genetic abnormalities, del(17p) is considered a key prognostic factor in MM (Rees et al. 2024). Although detection of *TP53*mut using next-generation sequencing (NGS) is still not part of many studies (Rees et al. 2024; Petrackova et al. 2020), new International Myeloma Society/International Myeloma Working Group (IMS/IMWG) Consensus 2025 recommends investigation of *TP53* mutations as part of routine practice (Avet-Loiseau et al. 2025). Moreover, data have demonstrated that multi-hit *TP53*, defined as a combination of del(17p) with *TP53*mut, or the presence of at least two *TP53*muts, is associated with less favourable prognosis, even in the era of novel agents (Sreedharanunni et al. 2024).

In this real-world study, we aimed to explore the impact of multi-hit *TP53* compared with other HR abnormalities and their co-occurrence on the survival of patients with MM treated with novel agents.

## Methods

### Patients’ characteristics and study design

The real-world cohort consisted of 204 patients diagnosed with MM according to the International Myeloma Working Group criteria (Rajkumar et al. 2014) who were diagnosed and treated between October 2017 and September 2024; a median follow-up was 28 months (range 1–219). In total, 134 (65.7%) bone marrow (BM) aspirates were obtained from newly diagnosed patients who were treatment naïve (TN) and 70 (34.3%) BM aspirates from patients who were relapsed/refractory (a median of one prior therapy line, range 1–9). All patients were treated with novel agent combinations: proteasome inhibitor (PI) based (bortezomib and carfilzomib), monoclonal antibodies (MoAb; daratumumab and isatuximab), immunomodulatory drugs (lenalidomide, pomalidomide and thalidomide) and bispecific antibodies (elranatamab and teclistamab). In addition, 43 patients received novel drugs with high-dose therapy with the support of autologous stem cell transplantation (ASCT). A comprehensive review based on medical records was conducted to collect demographic, clinical and genetic data related to MM. For 35 patients with MM, paired BM aspirates collected at progression were available (Supplementary Figure S1).

Patients were divided into three groups according to their genetic abnormalities: 1) HR with multi-hit *TP53* (subsequently referred to as multi-hit *TP53*), which included patients with concomitant del(17p) and *TP53*mut and/or carrying at least two *TP5*3muts; 2) HR without multi-hit *TP53* (subsequently referred to as HR); and 3) standard risk (SR). Subanalysis was performed in the HR subgroup based on the number of HR genetic abnormalities (1 vs ≥ 2 HR abnormalities) and HR with/without mono-hit *TP53* (i.e. only del(17p) or one *TP53*mut).

All patients provided written informed consent to sample and data collection for the purpose of this study, which was conducted in accordance with the Helsinki Declaration and approved by the Ethics Committee.

### Genetic analyses

A combination of FISH with immunophenotyping, called fluorescence-immunophenotyping and interphase cytogenetics as a tool for investigation of neoplasms (FICTION), was used to assess the cytogenetic abnormalities using following probes: LSI RB1 (Abbott Molecular, IL, USA), SPEC IGH, SPEC CKS1B/CDKN2C, TP53/c17, CCND1/IGH, FGFR3/IGH (Zytovision, Bremerhaven, Germany), XL MAF/IGH, CCND3/IGH, MAFB/IGH (MetaSystems, Altlussheim, Germany) and centromeric probes for chromosomes 7, 9, 11 and 15 (Cytocell, Cambridge, United Kingdom) as reported previously (Kriegova et al. 2021). A threshold of 10% was used as the cut‐off for translocations and 20% for numerical aberrations.

The full coding sequence of the *TP53* gene (exons 2–10 including 2 bp intronic overlaps, 5′ and 3′UTRs; NM_000546) was analysed from enriched CD138‐positive cells, as reported previously (Petrackova et al. 2020). Amplicon-based libraries were sequenced as paired-end on MiSeq (2 × 151, Illumina) with minimum target read depths of 5,000x. The limit of detection of *TP53*mut was set up to 1%, and the variants in the range 1–3% were confirmed by replication. After mapping to the human reference genome (GRCh38) using the Burrows–Wheeler Aligner–MEM algorithm (version 0.7.19, http://bio-bwa.sourceforge.net/), variant calling was performed using the Genome Analysis Toolkit (version 3.8, https://software.broadinstitute.org/gatk/): Unified Genotyper, Haplotype Caller, and MuTect (version 1.1.7). All detected sequence variants were manually checked using Integrative Genomics Viewer and annotated using clinical databases/tools (COSMIC, ClinVar, Ensemble Variant Effect Predictor tool). Only pathogenic or likely pathogenic variants were reported as assessed by ACMG criteria (Richards et al. 2015).

### Statistical analyses

The primary endpoint included the impact of HR genetic abnormalities on the progression-free survival (PFS) and overall survival (OS) of patients with MM. PFS was defined as the time between the start of treatment and disease progression or death. OS was defined as the time from diagnosis to the date of the last follow-up (censoring) or date of death. Event-free patients were censored in the analysis. The Kaplan–Meier model was used to represent the survival data, which were compared using the log-rank test. Relative risks (RRs) with 95% confidence intervals (CIs) are presented for the outcomes tested. Multivariable Cox hazards regression models were used to estimate associations of factors with time to event outcomes. Continuous laboratory and clinical variables are presented as medians and compared between groups using Mann–Whitney U test for two groups and the Kruskal–Wallis test for three or more groups. Categorical laboratory and clinical variables are compared between groups using Fisher's exact test. No adjustment for multiple comparisons was performed. A p-value of 0.05 was considered statistically significant. All statistical analyses were performed using Prism (GraphPad Software v10.2.3; Boston, MA, USA) or RStudio Software v2025.05.01 (Boston, MA, USA). GraphPad and Windows PowerPoint software were used for graphical representations of statistical analyses.

## Results

### Patient characteristics

Of the 204 patients with MM, twenty-four patients (11.9%) harboured multi-hit *TP53* aberrations, 108 (52.9%) belonged to HR group and 72 (35.2%) to SR group. For details on the demographic and clinical characteristics and treatment with novel agents see Table 1 and Supplementary Table S1.

**Table 1:** Clinical characteristics of enrolled patients with multiple myeloma and its subgroups

Characteristics	All cases^a^ (*n* = 204)	Multi-hit *TP53* (*n* = 24)	HR (*n* = 108)	SR (*n* = 72)
**Age (years), median**	69 (34–89)	69 (41–83)	69 (34–89)	69 (34–89)
**Sex, n (%)**				
Female	97 (47.5)	13 (54.2)	52 (48.1)	32 (44.4)
Male	107 (52.5)	11 (45.8)	56 (51.9)	40 (55.6)
**ISS stage, n (%)**				
Stage 1	62 (30.4)	5 (20.8)	24 (22.2)	33 (45.8)
Stage 2	49 (24.0)	7 (29.2)	28 (25.9)	14 (19.4)
Stage 3	89 (43.6)	12 (50.0)	54 (50.0)	23 (31.9)
NA	4 (2.0)	-	2 (1.9)	2 (2.8)
**Ig subtype, n (%)**				
Light chain only	39 (19.1)	4 (16.7)	17 (15.7)	18 (25.0)
IgA	43 (21.1)	4 (16.7)	29 (26.9)	10 (13.9)
IgG	120 (58.8)	16 (66.7)	61 (56.5)	43 (59.7)
IgD	1 (0.5)	-	1 (0.9)	-
NA	1 (0.5)	-	-	1 (1.4)
**Laboratory parameters, median (min–max)**				
White blood cell counts (10^9^/l)	5.46 (0.99–11.75)	4.30 (2.16–9.96)	5.42 (2.13–11.38)	5.96 (0.99–11.75)
Red blood cell counts (10^12^/l)	3.31 (2.10–5.34)	3.13 (2.25–4.10)	3.24 (2.36–4.84)	3.58 (2.10–5.34)
Haemoglobin (g/l)	107.0 (72.0–163.0)	100.5 (75.0–126.0)	105.5 (72.0–146.0)	115.0 (74.0–163.0)
Thrombocytes (10^9^/l)	186 (12–366)	168 (12–336)	165.5 (28–348)	207 (92–366)
Lymphocyte counts (10^9^/l)	1.38 (0.32–3.63)	0.88 (0.55–2.63)	1.30 (0.36–3.25)	1.45 (0.32–3.63)
Monocyte counts (10^9^/l)	0.48 (0.04–1.04)	0.46 (0.08–0.99)	0.47 (0.05–0.97)	0.50 (0.04–1.04)
Neutrophil counts (10^9^/l)	3.21 (0.54–8.46)	2.70 (0.83–6.41)	3.21 (0.69–8.05)	3.52 (0.54–8.46)
Eosinophil counts (10^9^/l)	0.08 (0.01–0.28)	0.03 (0.01–0.21)	0.08 (0.01–0.28)	0.08 (0.01–0.28)
Basophil counts (10^9^/l)	0.02 (0.01–0.08)	0.02 (0.01–0.08)	0.02 (0.01–0.06)	0.02 (0.01–0.04)
Urea (mmol/l)	6.20 (0.98–21.10)	6.90 (3.30–19.50)	6.50 (2.00–21.10)	5.75 (0.98–13.00)
Creatinine (µmol/l)	81 (39–274)	99 (53–274)	82 (44–240)	79 (39–123)
β2-microglobulin (mg/l)	2.96 (0.41–9.78)	5.68 (2.27–8.30)	3.50 (0.41–9.78)	2.42 (1.41–4.98)
Uric acid (µmol/l)	356 (83–660)	366 (235–456)	365 (159–660)	342 (83–585)
Bilirubin (µmol/l)	7 (2–17)	7 (3–12)	7 (3–16)	7 (2–17)
ALT (µkat/l)	0.39 (0.15–1.21)	0.38 (0.15–0.68)	0.39 (0.16–1.21)	0.37 (0.15–1.08)
AST (µkat/l)	0.37 (0.13–0.93)	0.38 (0.13–0.78)	0.34 (0.13–0.90)	0.42 (0.13–0.93)
ALP (µkat/l)	1.17 (0.44–2.49)	1.30 (0.61–2.29)	1.14 (0.44–2.21)	1.22 (0.54–2.49)
GGT (µkat/l)	0.47 (0.12–2.39)	0.64 (0.19–2.39)	0.44 (0.12–2.27)	0.50 (0.19–1.54)
LDH (µkat/l)	3.19 (1.10–5.86)	3.30 (1.54–5.14)	3.00 (1.10–5.86)	3.34 (1.66–5.15)
Total protein (g/l)	75.15 (43.30–119.90)	69.50 (51.40–99.40)	78.10 (45.30–119.90)	73.40 (43.30–108.90)
Albumin (g/l)	39.05 (21.50–52.50)	39.00 (26.00–44.30)	38.55 (21.50–48.00)	40.00 (27.20–52.50)
**Genetic aberrations, n (%)**				
t(4;14)	26 (12.7)	2 (8.3)	24 (22.2)	-
t(14;16)	8 (3.9)	1 (4.2)	7 (6.5)	-
1q21 gain/amp	91 (44.6)	13 (54.2)	78 (72.2)	-
del(17p)	35 (17.6)	15 (62.5)	20 (18.5)	-
del(1p)	26 (12.8)	4 (16.7)	22 (20.4)	-
t(11;14)	35 (17.6)	5 (20.8)	14 (13.0)	16 (22.2)
Hyperdiploidy	107 (52.5)	13 (54.2)	50 (46.3)	44 (61.1)
* TP53*mut	32 (15.7)	24 (100.0)	8 (7.4)	-
1 HR abnormality/≥ 2 HR abnormalities	62/70 (47.0/53.0)	0/24 (0.0/100.0)	62/46 (57.4/42.6)	-
**Treatment lines, n (%)**				
TN/1/2/≤ 3	134/37/11/22 (65.7/18.1/5.4/10.8)	6/6/5/7 (25.0/25.0/20.8/29.2)	75/18/5/10 (69.4/16.7/4.6/9.3)	53/13/1/5 (73.6/18.1/1.4/6.9)
**Treatment regimen, n (%)**				
PI based (bortezomib/carfilzomib)	98 (48.0) 88/10 (89.8/10.2)	8 (33.3) 5/3 (62.5/37.5)	56 (51.9) 52/4 (92.9/7.1)	34 (47.2) 31/3 (91.2/8.8)
ASCT	43 (21.1)	3 (12.5)	21 (19.4)	19 (26.4)
MoAb based (daratumumab/isatuximab)	41 (20.1) 29/12 (70.7/29.3)	10 (41.7) 9/1 (90.0/10.0)	19 (17.6) 11/8 (57.9/42.1)	12 (16.7) 9/3 (75.0/25.0)
Bispecfic antibodies (elranatamab/teclistamab)	11 (5.4) 7/4 (63.6/36.4)	1 (4.2) -/1 (0.0/100.0)	7 (6.5) 4/3 (57.1/42.9)	3 (4.2) 3/0 (100.0/0.0)
IMID based (lenalidomide/ pomalidomide/thalidomide)	11 (5.4) 7/3/1 (63.6/27.3/9.1)	2 (8.3) 1/1/0 (50.0/50.0/0.0)	5 (4.6) 3/2/0 (60.0/40.0/0.0)	4 (5.6) 3/0-/1 (75.0/0.0 /25.0)

^**a**^Patients are included only once

*ALP* Alkaline phosphatase, *ALT* Alanine aminotransferase, *ASCT* Autologous stem cell transplantation, *AST* Aspartate aminotransferase, *FLC* Free light chains, *GGT* Gamma-glutamyl transferase, *HR* High-risk, *IG* Immunoglobulin, *IMID* Immunomodulatory drug, *ISS* International staging system, *LDH* Lactate dehydrogenase, *MoAb* Monoclonal antibody, *NA* Not available, *PI* Proteasome inhibitor, *SR* Standard risk, *TN* Treatment-naïve

In the HR group, 62 patients (57.4%) had one HR abnormality: gain/amp 1q was detected in 38 patients (61.3%), del(17p) in 12 (19.3%), *TP53*mut in 5 (8.1%) and t(4;14) in 5 (8.1%); one patient (1.6%) had only t(14;16) and one (1.6%) had only del(1p32). Moreover, 46 patients (42.6%) had ≥ 2 co-occurring HR abnormalities. In the multi-hit *TP53* group, 9 patients (37.5%) had no other HR abnormality, 10 patients (41.7%) had one HR abnormality (8 patients with gain/amp 1q, 1 patient with t(4;14) and 1 patient with t(14;16) and 5 patients (20.8%) had two co-occurring other HR abnormalities. Of the patients with multi-hit *TP53* abnormalities, 87.5% and 12.5% were classified as patients with triple-hit and double-hit HR abnormalities (Rajkumar 2024), respectively.

Without *TP53*mut NGS analysis, 3.9% (8/204) of patients would have been misclassified into the SR group. Based on NGS, 20.8% (5/24) of patients were assigned to the multi-hit *TP53* group, as they carried ≥ 2 *TP53*muts and had no other HR abnormalities and 2.7% (3/108) to HR group, as they carried one *TP53*mut and had no other HR abnormality. The *TP53*mut, type, variant allele frequency (VAF), cancer clonal fraction and positions of the detected variants are listed in Supplementary Tables S2–S5, Figure S2. The majority of *TP53*mut detected were missense (80.0%), while nonsense, frameshift, and splice-site variants were relatively rare (4.6%, 7.7%, 7.7%, respectively; Supplementary Table S4). *TP53*mut were predominantly located within the DNA-binding domain (80.0%) and proline-rich domain (6.2%; Supplementary Figure S2).

### Patients with multi-hit *TP53* have the shortest survival

Aberrations of the *TP53* gene were detected in 52 (25.5%) patients. Of these, multi-hit *TP53* was identified in 24 (46.2%) patients. In the multi-hit *TP53* group, 15 (62.5%) patients exhibited concurrent del(17p) and ≥ 1 *TP53*mut and 9 (37.5%) had ≥ 2 *TP53*mut. To identify independent HR genetic abnormalities predicting short survival, Cox proportional hazard analysis was performed. Of all HR abnormalities, multi-hit *TP53* was a significant predictor of shorter OS (hazard ratio: 2.84, 95% CI: 1.29–6.23, *p* = 0.009; Supplementary Figure S3). Neither only del(17p) nor mono-hit *TP53*mut were significantly associated with reduced OS. Of the other clinical and laboratory characteristics included in the Cox analysis, only treatment other than ASCT was associated with reduced OS (Supplementary Figure S3).

**Fig. 1:**
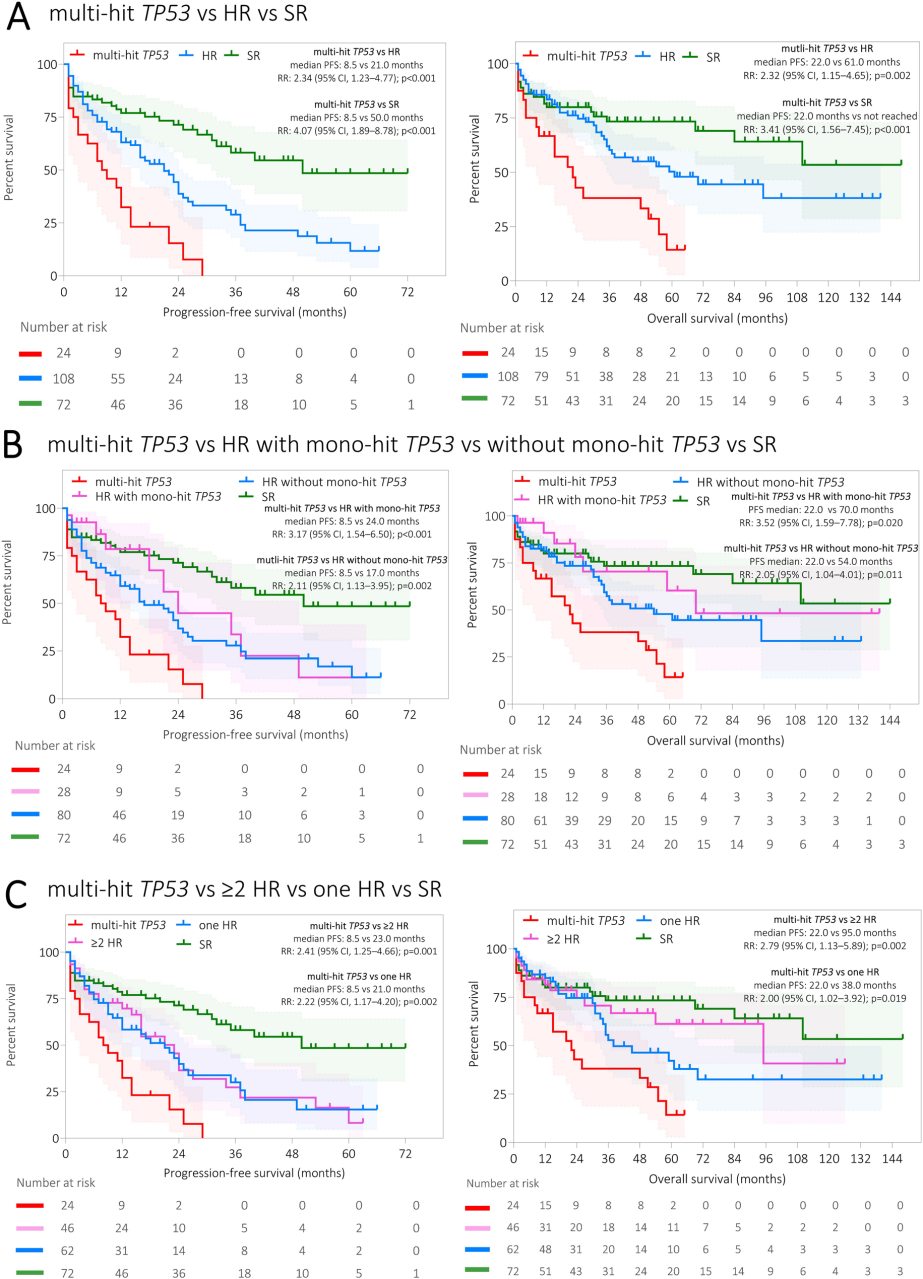
Progression-free survival (PFS) and overall survival (OS) of patients with MM with multi-hit *TP53*. **A** Patients with multi-hit *TP53* compared with the HR and SR groups (left panel PFS, right panel OS). **B** Patients with multi-hit *TP53* compared with patients with mono-hit *TP53* aberrations, patients who are HR without *TP53* aberrations and SR. **C** Patients with multi-hit *TP53* compared with ≥ 2 HR abnormalities, one HR abnormality and SR. The median PFS/OS (months) and 95% CI are mentioned in each graph. Mono-hit *TP53* is defined as patient with only del(17p) or one *TP53*mut. The distributions of OS and PFS were estimated by the Kaplan–Meier method. The log-rank test was used to determine statistically significant differences between the survival of different subgroups of patients. The Kaplan–Meier analysis was conducted in RStudio. Number of patients in particular subgroups are presented below the Kaplan–Meier curve. Abbreviations: CI: confidence interval; HR: high risk; MM: multiple myeloma; OS: overall survival; PFS: progression-free survival; SR: standard risk

Patients with multi-hit *TP53* had the shortest PFS and OS compared with the HR group (median PFS: 8.5 vs 21.0 months, RR: 2.34, 95% CI: 1.23–4.77, *p* < 0.001; median OS: 22.0 vs 61.0 months, RR: 2.32, 95% CI: 1.15–4.65, *p* = 0.002; Fig. 1) and the SR group (median PFS: 8.5 vs 50.0 months, RR: 4.07, 95% CI: 1.89–8.78, *p* < 0.001; median OS: 22.0 months vs not reached, RR: 3.41, 95% CI: 1.56–7.45, *p* < 0.001; Fig. 1). Patients with multi-hit *TP53* also had shorter PFS and OS than those with mono-hit *TP53* (only del(17p) or only *TP53*mut; median PFS: 8.5 vs 24.0 months, RR: 3.17, 95% CI: 1.54–6.50, *p* < 0.001; median OS: 22.0 vs 70.0 months, RR: 3.52, 95% CI: 1.59–7.78, *p* = 0.020; Fig. 1) and patients who were HR without *TP53* aberrations (median PFS: 8.5 vs 17.0 months, RR: 2.11, 95% CI: 1.13–3.95, *p* = 0.002; median OS: 22.0 vs 54.0 months, RR: 2.05, 95% CI: 1.04–4.01, *p* = 0.011; Fig. 1). In the mono-hit *TP53* group, 20 (71.4%) patients exhibited only del(17p) and 8 (28.6%) had one *TP53*mut. Patients with multi-hit *TP53* with a co-occurrence of other HR abnormalities did not differ in PFS to those with multi-hit *TP53* and without other HR abnormalities (*p* = 0.900).

Subsequently, the comparison of multi-hit *TP53* with ≥ 2 other HR abnormalities and one HR revealed that multi-hit *TP53* had shortest PFS and OS among the groups compared (multi-hit *TP53* vs ≥ 2 HR: median PFS: 8.5 vs 23.0 months, RR: 2.41, 95% CI: 1.25–4.66, *p* = 0.001; median OS: 22.0 vs 95.0 months, RR: 2.79, 95% CI: 1.13–5.89, *p* = 0.002; multi-hit *TP53* vs one HR: median PFS: 8.5 vs 21.0 months, RR: 2.22, 95% CI: 1.17–4.20, *p* = 0.002; median OS: 22.0 vs 38.0 months, RR: 2.00, 95% CI: 1.02–3.92, *p* = 0.019; Fig. 1). No difference in PFS (*p* = 0.807) and OS (*p* = 0.185; Fig. 1) was observed between patients with ≥ 2 other HR abnormalities and patients with one HR.

In major treatment groups (PI based, MoAb based), patients with multi-hit *TP53* consistently had inferior PFS (Supplementary Table S6 and S7). Patients with multi-hit *TP53* treated with PI based therapy had the shortest PFS compared to patients with HR (median PFS: 5.5 vs 21.0 months, RR: 2.35, 95% CI: 0.70–7.91, *p* = 0.040) and SR (median PFS: 5.5 months vs not reached, RR: 3.53, 95% CI: 0.87–14.31, *p* = 0.004; Supplementary Figure S4). Within MoAb based therapy, patients with multi-hit *TP53* had shorter PFS compared to patients with HR (median PFS: 7.0 vs 23.0 months, RR: 2.32, 95% CI: 0.86–6.27, *p* = 0.034) and SR (median PFS: 7.0 vs 28.0 months, RR: 3.71, 95% CI: 1.21–11.37, *p* = 0.002; Supplementary Figure S4). As only three patients with multi-hit *TP53* received ASCT, comparison could not be performed. Nevertheless, patients with HR had shorter PFS compared to those with SR within patients who received ASCT (median PFS: 34.0 months vs not reached, RR: 5.02, 95% CI: 1.81–13.90, *p* = 0.005; Supplementary Figure S4).

### Patients with high-risk abnormalities with ≥ 2 prior therapies show less favourable prognosis similarly to multi-hit *TP53*

**Fig. 2:**
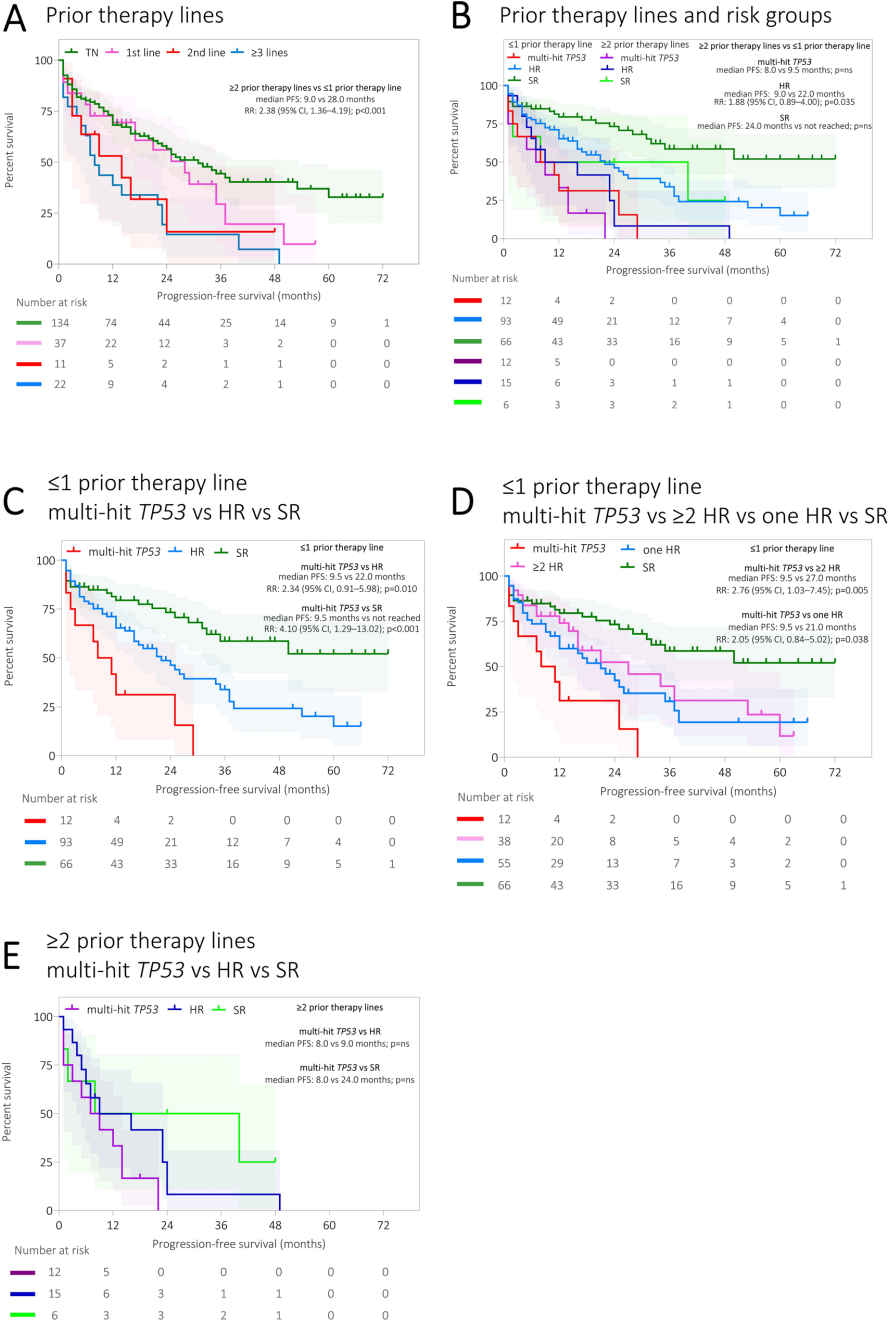
Progression-free survival (PFS) of patients with MM stratified by the number of prior therapy lines and genetic risk.** A** Patients with MM stratified by the number of prior therapy lines. **B** Patients with MM stratified by genetic risk and by the number of prior therapy lines. **C** Patients with multi-hit *TP53* compared with the HR and SR groups treated with ≤ 1 prior line of therapy. **D** Patients with multi-hit *TP53* compared with the ≥ 2 HR and one HR and SR groups treated with ≤ 1 prior line of therapy. **E** Patients with multi-hit *TP53* compared with patients in the HR and SR groups treated with ≥ 2 prior therapy lines. The median PFS/OS (months) and 95% CI are mentioned in each graph. The distributions of OS and PFS were estimated by the Kaplan–Meier method. The log-rank test was used to determine statistically significant differences between the survival of different subgroups of patients. The Kaplan–Meier analysis was conducted in RStudio. Number of patients in particular subgroups are presented below the Kaplan–Meier curve. Abbreviations: CI: confidence interval; HR: high risk; MM: multiple myeloma; PFS: progression-free survival; SR: standard risk; TN: treatment naïve

We were interested in whether PFS is dependent on the number of prior treatment lines in patients with MM. Because newly diagnosed patients did not differ in PFS from those with one prior therapy line (*p* = 0.299, Fig. 2), we grouped these patients for further analysis. In a whole cohort, patients with ≥ 2 prior therapy lines had shorter PFS than patients with ≤ 1 prior therapy line (median PFS: 9.0 vs 28.0 months, RR: 2.38, 95% CI: 1.36–4.19, *p* < 0.001; Fig. 2), irrespective of genetic risk. In the multi-hit *TP53* group, no difference in PFS was observed in patients with ≥ 2 prior therapy lines compared with patients with ≤ 1 prior therapy line (*p* = 0.442; Fig. 2). A reduced PFS in patients with ≥ 2 prior therapy lines compared with patients with ≤ 1 prior therapy line was observed in the HR group (median PFS: 9.0 vs 22.0 months, RR: 1.88, 95% CI: 0.89–4.00, *p* = 0.035; Fig. 2). Despite worse outcomes in patients in the SR group with ≥ 2 prior therapy lines compared with patients with ≤ 1 prior therapy line, significant difference in PFS was not observed (median PFS: 24.0 months vs not reached, RR: 2.22, 95% CI: 0.52–9.47, *p* = 0.125; Fig. 2). In patients with ≥ 2 prior therapy lines, no difference in PFS was detected between the multi-hit *TP53* and HR and SR groups (median PFS: 8.0 vs 9.0 vs 24.0 months, *p* = 0.140; Fig. 2).

For patients with ≤ 1 prior therapy line, multi-hit *TP53* (12; 7.0%) had the shortest PFS compared with the HR group (93; 54.4%) (median PFS: 9.5 vs 22.0 months, RR: 2.34, 95% CI: 0.91–5.98, *p* = 0.010; Fig. 2) and SR group (66; 38.6%) (median PFS: 9.5 months vs not reached, RR: 4.10, 95% CI: 1.29–13.02, *p* < 0.001; Fig. 2). Patients with multi-hit *TP53* and with ≤ 1 prior therapy line also had the shortest PFS compared with patients with ≥ 2 HR abnormalities (median PFS: 9.5 vs 27.0 months, RR: 2.76, 95% CI: 1.03–7.45, *p* = 0.005; Fig. 2) and one HR abnormality (median PFS: 9.5 vs 21.0 months, RR: 2.05, 95% CI: 0.84–5.02, *p* = 0.038; Fig. 2). When only TN patients were considered, the multi-hit *TP53* group had the shortest PFS compared with the HR group (median PFS: 9.5 vs 24.0 months, RR: 2.63, 95% CI: 0.65–10.72, *p* = 0.030) and the SR group (median PFS: 9.5 months vs not reached, RR: 3.92, 95% CI: 0.76–20.13, *p* = 0.003; Supplementary Figure S5).

### Accumulation of *TP53* aberrations in patients with multiple myeloma during treatment

**Fig. 3:**
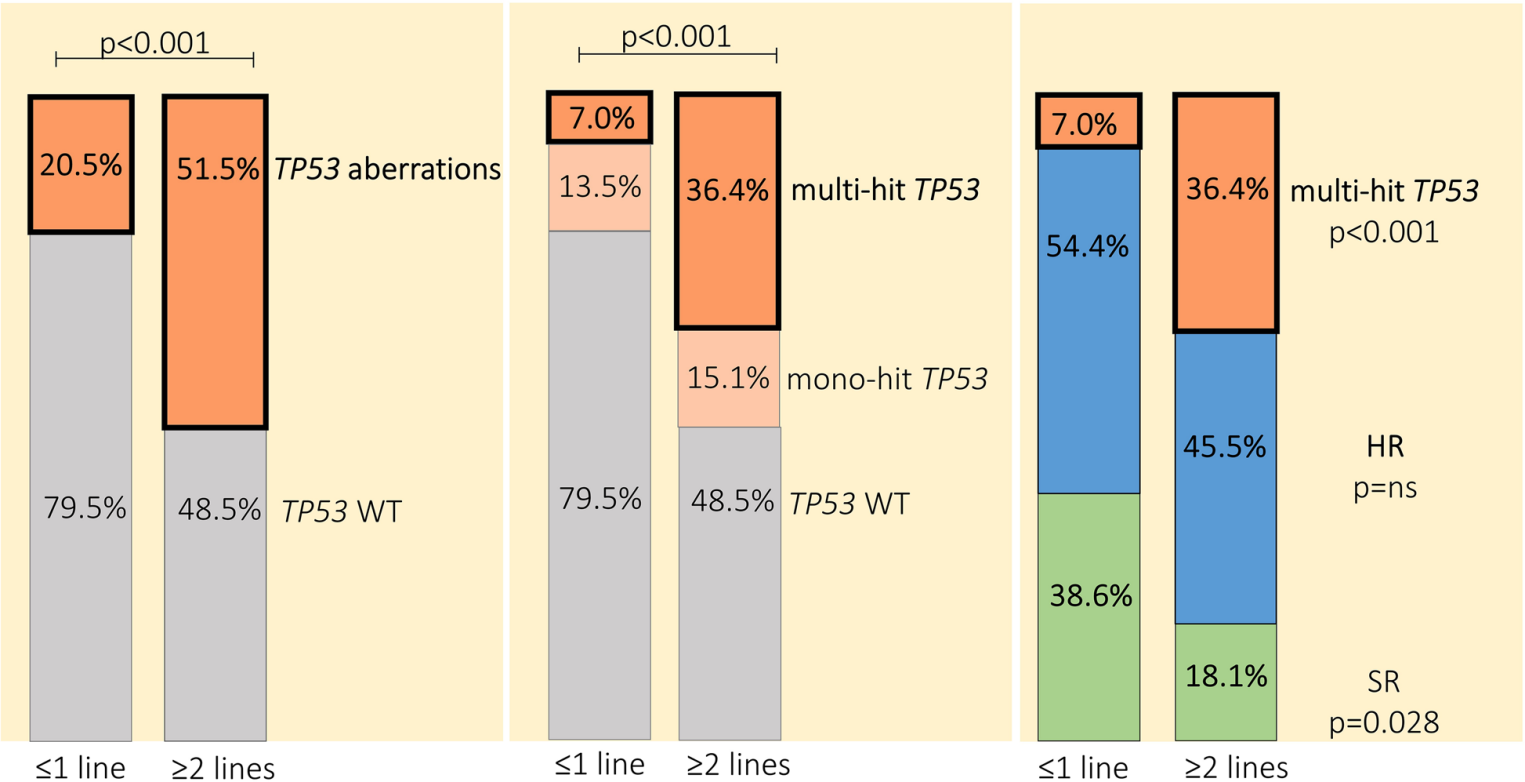
Accumulation of multi-hit *TP53* in patients with MM with ≥ 2 prior therapy lines. Frequency of multi-hit and mono-hit *TP53* aberrations in patients treated with ≤ 1 prior line of therapy (n = 171) and ≥ 2 prior therapy lines (n = 33). P-values were estimated using Fisher’s exact test. Abbreviations: HR: high risk; MM: multiple myeloma; SR: standard risk; TN: treatment naïve

The frequency of *TP53* aberrations was higher in patients with ≥ 2 prior therapy lines than in patients with ≤ 1 prior therapy line (51.5% vs 20.5%, RR: 2.52, 95% CI: 1.57–3.83, *p* < 0.001). Also, higher multi-hit *TP53* frequency was detected in patients with ≥ 2 prior therapy lines than in those with ≤ 1 prior therapy line (36.4% vs 7.0%, RR: 5.18, 95% CI: 2.54–10.25, *p* < 0.001; Fig. 3). Regarding other HR abnormalities, a higher frequency of t(4;14) abnormality was detected in patients with ≥ 2 prior therapy lines than in those with ≤ 1 prior therapy line (27.3% vs 9.9%, RR: 2.74, 95% CI: 1.32–5.40, *p* = 0.018; Supplementary Table S8). No difference was observed in the frequency of patients in the HR group with ≥ 2 prior therapy lines compared to patients with ≤ 1 prior therapy line (Fig. 3). Of note, compared with TN, a higher frequency of patients with multi-hit *TP53* was observed in relapsed/refractory patients (4.5% vs 25.7%, RR: 5.74, 95% CI: 2.46–13.51, *p* < 0.001; Supplementary Table S9).

Next, we considered the paired BM samples (baseline and progression) available for 35 patients with MM. At baseline sampling, *TP53* aberrations were present in nine patients (25.7%), three with del(17p) and six with *TP53*mut. The majority (83.0%) of patients with *TP53*mut at baseline exhibited higher VAF in the *TP53*mut at relapse, and 50% acquired additional *TP53*muts at relapse (Supplementary Table S5). For eight patients without *TP53* aberration at baseline, six acquired *TP53*mut and two del(17p) at relapse (Supplementary Table S10). No difference was observed between number of patients who acquired *TP53* aberrations after receiving ASCT (22.2%) compared to other therapies (33.3%; *p* = 0.676).

### Blood counts and laboratory parameters in multi-hit *TP53* multiple myeloma

**Fig. 4:**
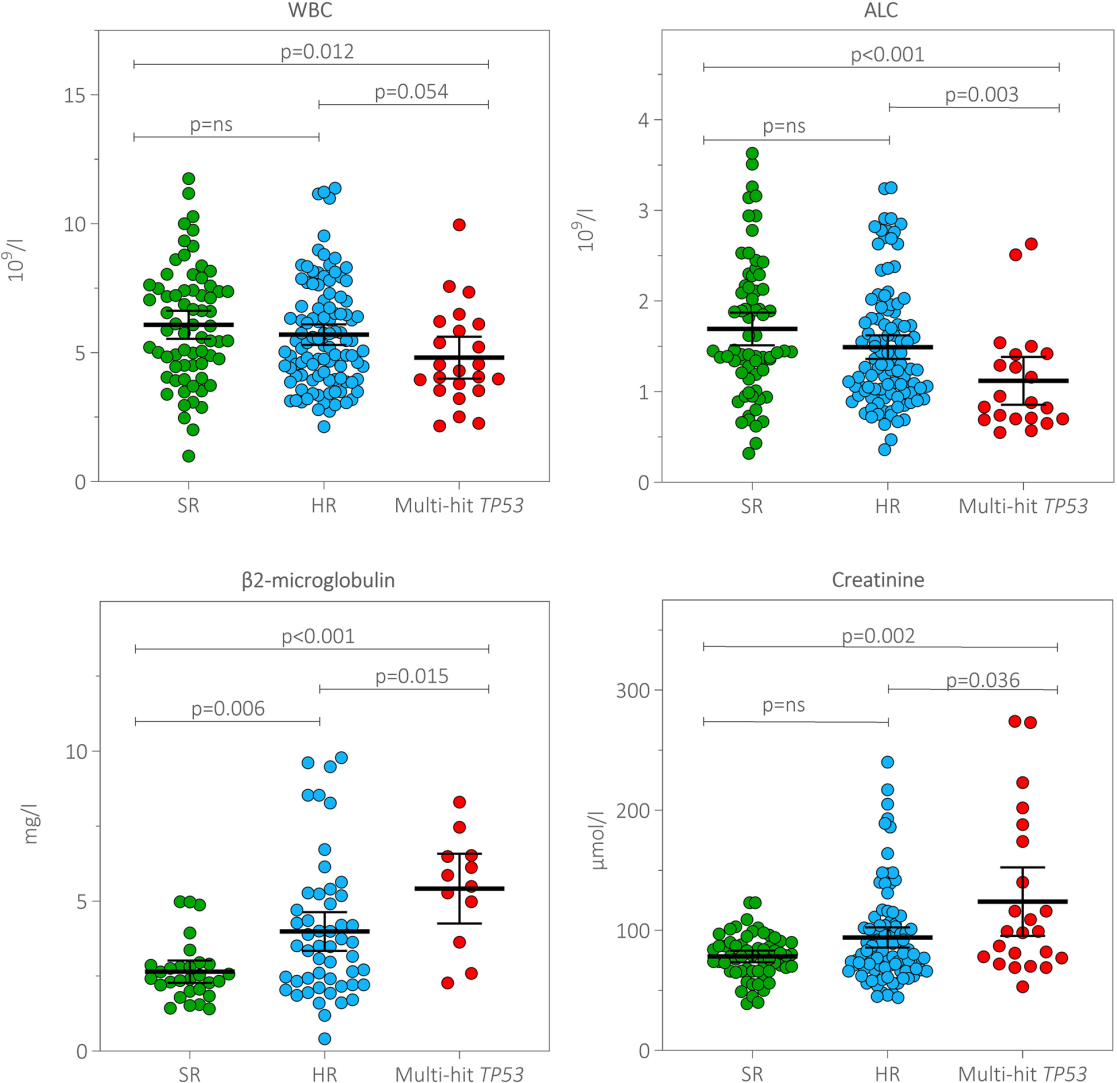
Distribution of blood counts, creatinine and β2-microglobulin serum levels in patients with MM within the multi-hit *TP53*, HR and SR groups. The Mann–Whitney U test was used to estimate *p*-values using RStudio. Group means are indicated by horizontal bars; error bars indicate 95% CIs. Abbreviations: ALC: absolute lymphocyte count; CI: confidence interval; HR: high-risk myeloma; MM: multiple myeloma; SR: standard-risk myeloma; WBC: white blood cell count

Next, we investigated the blood counts and laboratory parameters in patients in the multi-hit *TP53*, HR and SR groups. Among the three groups, patients with multi-hit *TP53* had the lowest white blood cell (median 10^9^/l: 4.30 vs 5.42 vs 5.96, *p* ≤ 0.054) and lymphocyte (median 10^9^/l: 0.88 vs 1.30 vs 1.45, *p* ≤ 0.003; Fig. 4) counts, with all values being within physiological values. Moreover, patients with multi-hit *TP53* had the highest serum levels of β2-microglobulin (median mg/l: 5.68 vs 3.50 vs 2.42, *p* ≤ 0.015) and creatinine (median µmol/l: 99.00 vs 82.00 vs 79.00, *p* ≤ 0.036; Fig. 4).

## Discussion

Despite therapeutic advances in MM, a subset of patients continues to experience disease progression, highlighting the need for refined risk stratification and individualised treatment approaches (Rees et al. 2024; Kaiser et al. 2025; Abu Za’nouneh et al. 2023). The present study provides a real-world analysis addressing the impact of different HR genetics and their co-occurrence, particularly the impact of multi-hit *TP53* on PFS, OS and the blood signature of patients with MM.

**Fig. 5:**
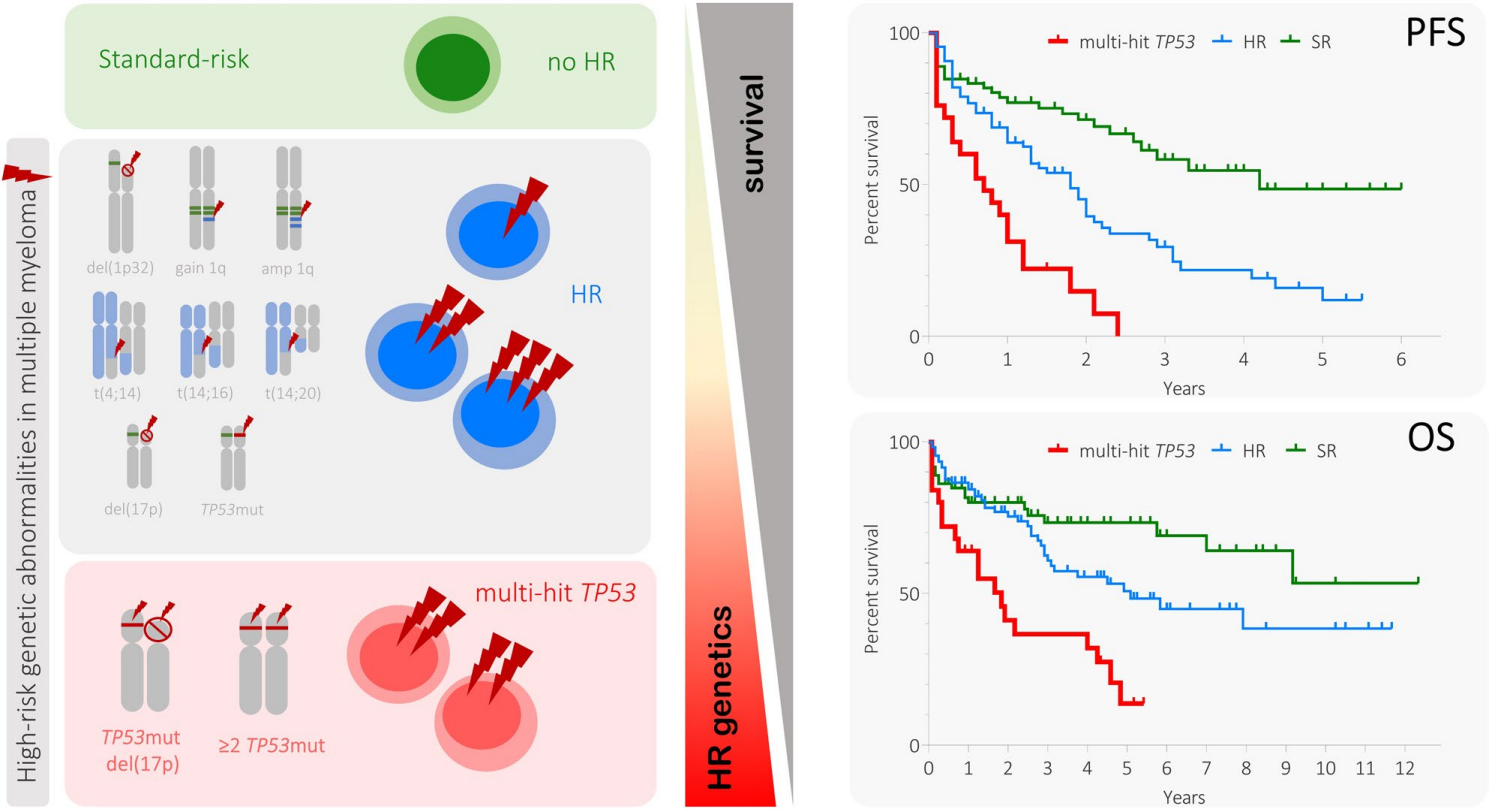
Results study overview. Abbreviations: HR: high risk, OS: overall survival, PFS: progression-free survival

A myriad of prognostic factors has been identified and incorporated into risk stratification models, but they still fail to identify many patients with MM developing early relapse (Rees et al. 2024). Evidence exists regarding the ultra-HR group with ≥ 2 HR genetic abnormalities (Rajkumar 2024; Rees et al. 2024), but it remains unclear whether specific HR abnormalities and their co-occurrence are less favourable than others. We assessed the impact of HR genetic abnormalities, such as t(4;14), t(14;16), t(14;20), gain/amp 1q, del(1p), del(17p) and *TP53*mut, and their co-occurrences in a real-world cohort, revealing that patients with a multi-hit *TP53* constitute the poorest prognostic group within the whole MM cohort. Patients with multi-hit *TP53* had very short PFS, independently on a number of therapy lines and major therapy strata. The relative risk of early progression in patients with multi-hit *TP53* is almost three times higher than that of patients with HR abnormalities and four times higher than that of patients in the SR group. Multi-hit *TP53* had the shortest survival within all the risk groups (multi-hit *TP53* vs HR vs SR; median PFS: 8.5 vs 21.0 vs 50.0 months; median OS: 22.0 vs 61.0 months vs not reached, respectively). Importantly, multi-hit *TP53* had the shortest survival when compared with the co-occurrence of ≥ 2 other HR genetic abnormalities (median PFS: 8.5 vs 23.0 months; median OS: 22.0 vs 95.0 months), a poor prognostic factor nominated by several studies (Fig. 5) (Rajkumar 2024; Rees et al. 2024; Kaiser et al. 2025). Our results are consistent with previous studies, including CoMMpass study (Ramón et al. 2022), which show that patients with multi-hit *TP53* and patients with biallelic *TP53* inactivation have inferior PFS and OS, but unlike our analysis, none of them provided a comparison with other HR abnormalities (Sreedharanunni et al. 2024; Martello et al. 2022; Walker et al. 2019).

Regarding functional impact of multi-hit *TP53*, a study using a MM cell line model observed abolished p53 activity and increased resistance to genotoxic drugs in cells harbouring two *TP53* hits, compared to cells with one or two wild-type *TP53* alleles (Munawar et al. 2019). *TP53* suppresses tumor development by regulating genes involved in apoptosis, cell-cycle arrest, and DNA repair, and to perform its transcription factor function, four p53 molecules self-assemble on DNA to form a tetramer. While some *TP53* mutations cause loss of the p53 protein, most are missense substitutions in the DNA-binding domain that impair protein function (Joerger et al. 2025; Wang et al. 2024). Mutant p53 proteins have been proposed to promote malignancy through three processes: (i) loss-of-function, i.e., the inability of mutant p53 to activate the expression of genes that are transcriptionally activated by wild type (WT) p53 to suppress tumorigenesis; (ii) exerting a dominant-negative effect over the WT protein through the formation of mixed tetramers with weakened DNA-binding; and (iii) gain-of-function, which is mediated by co-aggregation of mutant p53 with other transcriptional regulators, including the paralogs p63 and p73 (Joerger et al. 2025; Wang et al. 2024). In thermolabile p53 mutants, a series of aggregation-prone sequence motifs are exposed, driving aberrant interactions and cellular responses not normally affected by WT p53. For example, binding to p73 has been postulated as one possible gain-of-function mechanism of mutant p53, thereby reducing the possibility of a salvage pathway (Li and Prives 2007). In our study, distributions and types of *TP53*mut were in line with previously published results in MM (Sreedharanunni et al. 2024; Walker et al. 2019; Chin et al. 2017; Leroy et al. 2013).

We observed an increased frequency of *TP53* abnormalities in patients with ≥ 2 prior therapy lines, further supporting the concept that treatment pressure drives clonal evolution in MM, favouring the outgrowth of *TP53*-aberrant populations (Sreedharanunni et al. 2024; Martello et al. 2022; Chin et al. 2017; Jovanović et al. 2019; Kortüm et al. 2016). Importantly, multi-hit *TP53* was 5 times more frequent in patients with ≥ 2 prior therapy lines compared to patients with less therapy lines. In our cohort, the same frequency of patients who acquired *TP53* aberrations after receiving ASCT was observed compared to other therapies. Notably, our analysis of longitudinal samples identified that the majority of patients with *TP53*mut at baseline exhibited higher VAF of the *TP53*mut at relapse, and half acquired additional *TP53*mut, as demonstrated also by others (Sreedharanunni et al. 2024; Martello et al. 2022). Considering that *TP53* alterations increase during progression of MM and are associated with drug resistance, it might be necessary to interact with *TP53* pathway to be able to significantly improve outcomes of MM patients, or at least develop treatment strategies that do not select *TP53* subclones (Jovanović et al. 2019). Based on ex vivo drug screening, MM with *TP53*mut may be targetable by approved inhibitors of mitosis, topoisomerase, HDAC, HSP90, IGF1R and PI3K/AKT/mTOR pathways (Tsallos et al. 2024). Another study revealed that a BH3 mimetics combination may be beneficial for patients with biallelic *TP53* disruption (Durand et al. 2024).

When we investigated the relationship between HR genetic abnormalities and treatment lines, patients with HR who had received ≥ 2 prior lines had shorter PFS than those with ≤ 1 prior therapy line (median PFS: 9.0 vs 22.0 months), having an equally detrimental effect on survival as multi-hit *TP53* (median PFS: 8.0 vs 9.5 months). As the study cohort included modest number of patients who received ≥ 2 prior lines, this result should be confirmed in larger cohorts. However, the influence of treatment lines on survival in MM has been already reported (Avet-Loiseau et al. 2010; Wang et al. 2023) and may be linked to the dysfunctional microenvironment (Visram et al. 2021 Mar 1).

Recent studies suggest that the co-occurrence of ≥ 2 HR (double-hit and triple-hit MM) genetic abnormalities form an ultra-HR group (Kaiser et al. 2025; Baysal et al. 2020; Shen et al. 2021). However, these studies have not assessed *TP53*mut status. In our cohort, the co-occurrence of ≥ 2 HR abnormalities, excluding multi-hit *TP53*, was not associated with reduced PFS and OS when compared with patients with one HR abnormality. By contrast, the co-occurrence of ≥ 2 HR abnormalities had longer PFS and OS than multi-hit *TP53* in MM. In our cohort, 87.5% and 12.5% of patients with multi-hit *TP53* could be classified as having triple-hit and double-hit HR abnormalities, respectively. These observations suggest that the shorter survival of double-hit and triple-hit MM is more likely attributed to the presence of underlying multi-hit *TP53* than to the co-occurrence of other multiple HR abnormalities. Future studies should confirm our observation in larger cohorts.

We further emphasise the importance of *TP53*mut assessment in MM. In our cohort, ~ 4% of the patients would have been misclassified into the SR group without NGS analysis of *TP53*mut. Moreover, without NGS, we would have missed all patients with multi-hit *TP53* that occurred at a frequency of ~ 7% in those with ≤ 1 prior therapy line and at ~ 36% in those with ≥ 2 prior therapy lines. Our data further support current IMS/IMWG consensus, which has updated MM prognostic classification and recommends that *TP53*mut, identified using NGS-based methods on CD138-positive/purified cells, should be included in the HR MM definition (Avet-Loiseau et al. 2025). Routine assessment of *TP53* mutational status in MM is particularly crucial for patients with multi-hit *TP53* and those with *TP53*mut but without del(17p), as they will not be correctly risk stratified and are thus less likely to be prioritised for novel therapies within trials or for earlier or optimised therapeutic intervention (Berdeja et al. 2021; Moreau et al. 2022; Munshi et al. 2021; Pasvolsky et al. 2023).

In examining the circulating immune microenvironment, we found that patients with multi-hit *TP53* had the lowest counts of white blood cells and lymphocytes and the highest serum creatinine and β2-microglobulin levels compared with patients in the HR and SR groups. A recent study evaluating 11,427 patients with MM found that lymphopenia at diagnosis and during treatment and follow-up was associated with inferior OS (Ferri et al. 2025). However, this study did not evaluate *TP53* and other HR abnormalities, and we may only hypothesise that, in some patients, the underlying lymphopenia might be associated with multi-hit *TP53* abnormalities. In addition, our patients with multi-hit *TP53* exhibited elevated levels of β2-microglobulin, a prognostic factor of poor prognosis, and creatinine, an indicator of renal impairment, when compared with the HR and SR groups. These findings further support the association of multi-hit *TP53* with more aggressive disease phenotypes.

This study has several limitations. Despite the modest size of the patient cohort, especially in group of patients with ≥ 2 prior therapies, this is a well-clinically characterised, real-world cohort of patients with the full spectrum of evaluated genetic HR abnormalities, including *TP53*mut. Moreover, the study covers patients with MM who are newly diagnosed as well as those who are relapsed/refractory, which enables us to evaluate the impact of treatment lines and the accumulation of *TP53* abnormalities. Our results are consistent with previously published results (Sreedharanunni et al. 2024; Ramón et al. 2022; Martello et al. 2022; Walker et al. 2019) but future studies on larger cohorts would strengthen our findings. In multi-hit *TP53* cases, we could not determine the allelic configuration (cis or trans) or whether the aberrations occurred within the same clone, as *TP53* mutations were identified by diagnostic NGS using short-read sequencing. We included all patients on novel agents, but a subanalysis of patients with bispecific antibodies could not be performed because only one patient had multi-hit *TP53*. Similarly, we were not able to evaluate the benefit of ASCT because of the small number of patients in the multi-hit *TP53* group treated using this modality. Furthermore, it cannot be excluded that patients treated with combination of one novel agent had the least favourable prognosis of all the diagnostic groups because they were clinically frail and had a higher risk of treatment-related toxicity.

This study demonstrates that patients with multi-hit *TP53* represent the poorest prognostic group in MM, even when compared with patients with ≥ 2 other HR genetic abnormalities and independently of the number of prior lines of therapy. The relative risk of early progression in patients with multi-hit *TP53* is almost three times higher than that of patients with other HR abnormalities and four times higher than that of patients in the SR group. The frequency of patients with multi-hit *TP53* increases in later disease stages even in the context of novel therapies. We also emphasize the importance of *TP53*mut assessment in routine practice and studies in MM. Our real-world study highlights the value of redefining HR stratification in MM and the need for novel therapeutic strategies for patients with multi-hit *TP53*.

## Supplementary Information

Supplementary Material 1.

## Data Availability

The datasets analysed during the current study are available from the corresponding author on reasonable request.
