# Supplementary material for: Multi-hit TP53 confers the poorest survival in multiple myeloma in the era of novel therapies
Source: Mol Med. 2025 Nov 29;32:3. doi: 10.1186/s10020-025-01392-2 (PMC12771809; doi:10.1186/s10020-025-01392-2)
Supplement: Supplementary file 1 — Supplementary Material 1. [file 10020_2025_1392_MOESM1_ESM.docx]

The Supplementary File

**Multi-hit *TP53* confers the poorest survival in multiple myeloma in the era of novel therapies**

Nesnadna R, Petrackova A, Minarik J, Latal V, Manakova J, Papajik T, Kriegova E*.*

**Tables:**

**Table S1** Clinical characteristics of enrolled patients with multiple myeloma divided to treatment-naïve and relapse/refractory.

**Table S2** Number of *TP53* mutations detected in each patient with MM.

**Table S3** The variant allele frequency (VAF) of *TP53* mutations in patients with MM (included paired samples).

**Table S4** Types of *TP53* mutations detected in our cohort of patients with MM (included paired samples).

**Table S5** Details of *TP53* mutations identified in patients with MM (included paired samples: referred as baseline and relapse).

**Table S6** Treatment regimens across risk groups stratified by treatment lines.

**Table S7** Distribution of treatment regimens across the risk groups.

**Table S8** The frequency of HR cytogenetic abnormalities in MM patients treated with ≤1 and ≥2 prior therapy lines.

**Table S9** The frequency of *TP53* aberrations, multi-hit *TP53* and mono-hit *TP53* aberrations in treatment-naïve and relapsed/refractory patients with MM.

**Table S10** Overview of *TP53* abnormalities in paired bone marrow samples analysed at baseline and at relapse/progression in MM patients.

**Figures:**

**Fig. S1** Flow diagram of analysed samples from patients with MM.

**Fig. S2** Lollipop plot representing the location of detected *TP53*mut in all analysed samples.

**Fig. S3** Multivariate Cox regression analysis of prognostic genetic abnormalities, treatment regimens and clinical parameters associated with overall survival in patients with MM.

**Fig. S4** Progression-free survival of patients with multiple myeloma treated with different therapeutic regimens and stratified by risk into multi-hit *TP53*, HR, and SR groups.

**Fig. S5** Progression-free survival of treatment-naïve patients with MM.

**Supplementary Table S1** Clinical characteristics of enrolled patients with multiple myeloma divided to treatment-naïve and relapse/refractory.

| Characteristics | All cases  (n=204) | TN  (n=134) | RR  (n=70) | *p* |
| --- | --- | --- | --- | --- |
| Age (years), median | 69 (34–89) | 69 (41–89) | 70 (34–83) | 0.630 |
| Sex, n (%) |  |  |  |  |
| Female/Male | 97 (47.5)/ 107 (52.5) | 53 (39.6)/ 81 (60.4) | 44 (62.9)/ 26 (37.1) | 0.002 |
| ISS stage, n (%) |  |  |  |  |
| Stage 1 | 62 (30.4) | 35 (26.1) | 27 (38.6) | 0.078 |
| Stage 2 | 49 (24.0) | 28 (20.9) | 21 (30.0) | 0.169 |
| Stage 3 | 89 (43.6) | 69 (51.5) | 20 (28.6) | 0.002 |
| NA | 4 (2.0) | 2 (1.5) | 2 (2.9) | - |
| Ig subtype, n (%) |  |  |  |  |
| Light chain only | 39 (19.1) | 28 (20.9) | 11 (15.7) | 0.455 |
| IgA | 43 (21.1) | 32 (23.9) | 11 (15.7) | 0.208 |
| IgG | 120 (58.8) | 74 (55.2) | 46 (65.7) | 0.178 |
| IgD | 1 (0.5) | - | 1 (1.4) | - |
| NA | 1 (0.5) | - | 1 (1.4) | - |
| Laboratory parameters, median (min–max) |  |  |  |  |
| White blood cell counts (10^9^/l) | 5.46 (0.99–11.75) | 5.76 (0.99–11.75) | 4.87 (2.13–9.96) | 0.024 |
| Red blood cell counts (10^12^/l) | 3.31 (2.10–5.34) | 3.16 (2.10–5.34) | 3.51 (2.62–5.25) | < 0.001 |
| Haemoglobin (g/l) | 107 (72–163) | 98 (72–157) | 116 (83–163) | < 0.001 |
| Thrombocytes (10^9^/l) | 186.0 (12.0–366.0) | 193.0 (16.0–366.0) | 181.5 (12.0–348.0) | 0.072 |
| Lymphocytes counts (10^9^/l) | 1.38 (0.32–3.63) | 1.41 (0.32–3.63) | 1.36 (0.55–3.02) | 0.560 |
| Monocyte counts (10^9^/l) | 0.48 (0.04–1.04) | 0.47 (0.04–0.99) | 0.49 (0.08–1.04) | 0.055 |
| Neutrophils counts (10^9^/l) | 3.21 (0.54–8.46) | 3.52 (0.54–8.46) | 2.81 (0.69–6.51) | 0.041 |
| Eosinophil counts (10^9^/l) | 0.08 (0.01–0.28) | 0.05 (0.01–0.28) | 0.08 (0.01–0.24) | 0.190 |
| Basophil count (10^9^/l) | 0.02 (0.01–0.08) | 0.02 (0.01–0.08) | 0.02 (0.01–0.08) | 0.880 |
| Urea (mmol/l) | 6.20 (0.98–21.10) | 6.50 (2.00–21.10) | 6.00 (0.98–16.60) | 0.004 |
| Creatinine (µmol/l) | 81 (39–274) | 83 (45–274) | 83 (39–273) | 0.360 |
| β2-microglobulin (mg/l) | 2.96 (0.41–9.78) | 2.96 (0.41–9.61) | 3.35 (1.41–9.78) | 0.490 |
| Uric acid (µmol/l) | 356 (83–660) | 376 (145–660) | 340 (83–511) | 0.065 |
| Bilirubin (µmol/l) | 7 (2–17) | 6 (2–15) | 8 (3–17) | 0.001 |
| ALT (µkat/l) | 0.39 (0.15–1.21) | 0.40 (0.15–1.21) | 0.36 (0.15–1.05) | 0.370 |
| AST (µkat/l) | 0.37 (0.13–0.93) | 0.39 (0.13–0.89) | 0.36 (0.13–0.93) | 0.410 |
| ALP (µkat/l) | 1.17 (0.44–2.49) | 1.17 (0.44–2.42) | 1.18 (0.48–2.49) | 0.500 |
| GGT (µkat/l) | 0.47 (0.12–2.39) | 0.58 (0.12–2.39) | 0.38 (0.18–2.25) | 0.016 |
| LDH (µkat/l) | 3.19 (1.10–5.86) | 2.95 (1.10–5.86) | 3.29 (1.66–5.14) | 0.150 |
| Total protein (g/l) | 75.15 (43.30–119.90) | 81.80 (43.30–119.90) | 70.5 (52.60–115.50) | 0.002 |
| Albumin (g/l) | 39.05 (21.50–52.50) | 37.00 (21.50–52.50) | 42.00 (23.20–48.00) | < 0.001 |
| Genetic aberrations, n (%) |  |  |  |  |
| t(4;14) | 26 (12.7) | 13 (9.7) | 13 (18.6) | 0.713 |
| t(14;16) | 8 (3.9) | 7 (5.2) | 1 (1.4) | < 0.001 |
| 1q21 gain/amp | 91 (44.6) | 57 (42.5) | 34 (48.6) | 0.410 |
| del(17p) | 35 (17.2) | 19 (14.2) | 16 (22.9) | 0.119 |
| del(1p) | 26 (12.8) | 21 (15.7) | 5 (7.1) | 0.083 |
| t(11;14) | 35 (17.2) | 19 (14.2) | 16 (22.9) | 0.047 |
| Hyperdiploidy | 107 (52.5) | 77 (57.5) | 30 (42.9) | 0.119 |
| *TP53*mut | 32 (15.7) | 8 (6.0) | 24 (34.3) | < 0.001 |
| 1 HR abnormality / ≥2 HR abnormalities | 62/70  (47.0/53.0) | 44/37  (54.3/45.7) | 18/33  (35.3/64.7) | 0.338/0.008 |
| Treatment lines, n (%) |  |  |  |  |
| TN/1/2/≤3 | 134/37/11/22  (65.7/18.1/5.4/10.8) | 134/-/-/-  (100/-/-/-) | -/37/11/22  (-/52.9/15.7/31.4) | - |
| Treatment regimen, n (%) |  |  |  |  |
| PI based  (bortezomib/carfilzomib) | 98 (48.0)  88/10 (89.8/10.2) | 85 (63.4)  85/- (100.0/-) | 13 (18.6)  3/10 (23.1/76.9) | < 0.001 |
| ASCT | 43 (21.1) | 36 (26.9) | 7 (10.0) | 0.005 |
| MoAb based (daratumumab/isatuximab) | 41 (20.1)  29/12 (70.7/29.3) | 5 (3.7)  1/4 | 36 (51.4)  28/8 (77.8/22.2) | < 0.001 |
| Bispecfic antibodies  (elranatamab/teclistamab) | 11 (5.4)  7/4 (63.6/36.4) | 3 (2.2)  3/- (100.0/-) | 8 (11.4)  4/4 (50.0/50.0) | 0.006 |
| IMID based (lenalidomide/pomalidomide/thalidomide) | 11 (5.4)  7/3/1 (63.6/27.3/9.1) | 5 (3.7)  4/-/1(80.0/-/20.0) | 6 (8.6)  3/3/- (50.0/50.0/-) | 0.146 |

P-values were estimated using Fisher’s exact test and Mann–Whitney U test.

**Abbreviations:** ALP: alkaline phosphatase; ALT: alanine aminotransferase; ASCT: autologous stem cell transplantation; AST: aspartate aminotransferase; FLC: free light chains; GGT: gamma-glutamyl transferase; HR: high-risk; IG: immunoglobulin; IMID: immunomodulatory drug; ISS: international staging system; LDH: lactate dehydrogenase; MoAb: monoclonal antibody; NA: not available; PI: proteasome inhibitor; SR: standard risk; TN: treatment-naïve.

**Supplementary Table S2** Number of *TP53* mutations detected in patients with MM.

| ***TP53*mut, n** | **Patients, n** |
| --- | --- |
| 1 | 17 |
| 2 | 12 |
| ≥3 | 3 |

**Supplementary Table S3** The variant allele frequency (VAF) of *TP53* mutations in patients with MM (included paired samples).

| **VAF (%)** | **n** | **%** |
| --- | --- | --- |
| 1–10 | 37 | 56.9 |
| 11–20 | 7 | 10.8 |
| 21–30 | 6 | 9.2 |
| 31–40 | 4 | 6.2 |
| 41–50 | 2 | 3.1 |
| ˃ 50 | 9 | 13.8 |

**Supplementary Table S4** Types of *TP53* mutations detected in our cohort of patients with MM (included paired samples).

| **Type of mutation** | **n** | **%** |
| --- | --- | --- |
| missense | 52 | 80.0 |
| nonsense | 3 | 4.6 |
| frameshift | 5 | 7.7 |
| splice region | 5 | 7.7 |

**Supplementary Table S5** Details of *TP53* mutations identified in patients with MM (included paired samples: referred as baseline and relapse).

| **Patient** | **cDNA** | **Protein** | **Variant type** | **VAF** | **dbSNP/COSMIC** | **CCF*** |
| --- | --- | --- | --- | --- | --- | --- |
| P1_baseline | c.824G>A | p.C275Y | missense | 0.33 | rs863224451/COSV52661919 | 0.69 |
| P1_relapse | c.824G>A | p.C275Y | missense | 0.64 | rs863224451/COSV52661919 | 1 |
|  | c.273G>A | p.W91* | nonsense | 0.14 | rs876660548/COSV52673580 | 0.52 |
| P2_baseline | c.713G>C | p.C238S | missense | 0.02 | rs730882005/COSV52804264 | 0.06 |
| P2_relapse | c.713G>C | p.C238S | missense | 0.23 | rs730882005/COSV52804264 | 1 |
| P3_baseline | c.530C>T | p.P177L | missense | 0.04 | rs751477326/COSV52721872 | 0.16 |
| P3_relapse | c.530C>T | p.P177L | missense | 0.08 | rs751477326/COSV52721872 | 1 |
| P4_baseline | c.764T>C | p.I255T | missense | 0.02 | rs876659675/COSV52712740 | 0.09 |
|  | c.841G>A | p.D281N | missense | 0.04 | rs764146326/COSV52671054 | 0.03 |
| P4_relapse | c.527G>T | p.C176F | missense | 0.12 | rs786202962/COSV52661329 | 0.26 |
|  | c.638G>C | p.R213P | missense | 0.16 | rs587778720/COSV52693072 | 0.67 |
|  | c.535C>G | p.H179D | missense | 0.40 | rs587780070/COSV52668276 | 0.20 |
| P5_baseline | c.830G>T | p.C277F | missense | 0.04 | rs763098116/COSV52693726 | 0.11 |
| P5_relapse | c.830G>T | p.C277F | missense | 0.02 | rs763098116/COSV52693726 | 0.06 |
| P6_baseline | c.-29+2T>G | - | splice region | 0.03 | -/- | 0.12 |
| P6_relapse | c.-29+2T>G | - | splice region | 0.16 | -/- | 0.16 |
|  | c.743G>A | p.R248Q | missense | 0.08 | rs11540652/- | 0.13 |
|  | c.395A>T | p.K132M | missense | 0.07 | rs1057519996/- | 0.38 |
|  | c.775G>A | p.D259N | missense | 0.19 | -/- | 0.32 |
| P7_relapse | c.358A>G | p.K120E | missense | 0.26 | rs121912658/COSV52692352 | 0.60 |
| P8_relapse | c.733G>A | p.G245S | missense | 0.03 | rs28934575/COSV52661877 | 0.08 |
| P9_relapse | c.830G>A | p.C277Y | missense | 0.02 | rs763098116/COSV52826087 | 0.19 |
| P10_relapse | c.404G>T | p.C135F | missense | 0.03 | rs587781991/COSV52680475 | 1 |
| P11_relapse | c.503A>C | p.H168P | missense | 0.23 | -/COSV52676197 | 1 |
| P12_relapse | c.469G>C | p.V157L | missense | 0.17 | -/COSV52752314 | 0.44 |
|  | c.746G>C | p.R249T | missense | 0.06 | rs587782329/COSV52697169 | 0.14 |
| P13 | c.261_265insGGGCT | p.P89fs | frameshift | 0.51 | -/- | 1 |
| P14 | c.640C>T | p.H214Y | missense | 0.84 | -/- | 1 |
| P15 | c.1101-1G>A | - | splice region | 0.31 | rs876658982/COSV53196286 | 1 |
| P16 | c.786_787delAT | p.N263fs | frameshift | 0.09 | -/- | 0.22 |
|  | c.560-1G>A | - | splice region | 0.02 | rs1202793339/- | 0.05 |
| P17 | c.623A>G | p.D208G | missense | 0.01 | rs1464727668/COSV52744439 | 1 |
|  | c.248C>T | p.A83V | missense | 0.04 | rs867725248/COSV105030175 | 1 |
| P18 | c.856G>A | p.E286K | missense | 0.31 | rs786201059/- | 0.67 |
|  | c.379T>C | p.S127P | missense | 0.09 | -/- | 0.21 |
| P19 | c.517G>A | p.V173M | missense | 0.93 | rs876660754/COSV52677795 | 1 |
| P20 | c.1006G>T | p.E336* | nonsense | 0.46 | -/COSV52746809 | 1 |
|  | c.734G>A | p.G245D | missense | 0.48 | rs121912656/COSV52667838 | 1 |
| P21 | c.809T>C | p.F270S | missense | 0.61 | rs1057519986/COSV52676663 | 0.87 |
|  | c.743G>A | p.R248Q | missense | 0.04 | rs11540652/- | 0.06 |
| P22 | c.532C>G | p.H178D | missense | 0.03 | -/- | 0.06 |
|  | c.512A>G | p.E171G | missense | 0.04 | -/- | 0.17 |
|  | c.527G>A | p.C176Y | missense | 0.02 | -/- | 0.09 |
|  | c.722C>A | p.S241Y | missense | 0.02 | rs28934573/- | 0.04 |
|  | c.726C>G | p.C242W | missense | 0.02 | rs375874539/- | 0.04 |
|  | c.747G>T | p.R249S | missense | 0.04 | rs28934571/- | 0.05 |
|  | c.764T>A | p.I255N | missense | 0.04 | rs876659675/- | 0.08 |
|  | c.395A>T | p.K132M | missense | 0.08 | rs1057519996/- | 0.07 |
|  | c.746G>C | p.R249T | missense | 0.04 | rs587782329/COSV52697169 | 0.08 |
| P23 | c.475_476delGC | p.A159fs | frameshift | 0.97 | -/- | 1 |
| P24 | c.658T>A | p.Y220N | missense | 0.77 | rs530941076/COSV52700454 | 1 |
| P25 | c.1037A>G | p.E346G | missense | 0.02 | -/- | 0.17 |
| P26 | c.393C>A | p.N131K | missense | 0.03 | rs769270327/COSV53696738 | 0.13 |
| P27 | c.524G>A | p.R175H | missense | 0.09 | rs28934578/COSV52661038 | 0.21 |
| P28 | c.466_467insCCGCCCGGCACCC | p.R156fs | frameshift | 0.29 | -/- | 0.48 |
| P29 | c. 560-5_560-15delACTGATTGCTC | - | splice region | 0.15 | -/- | 0.07 |
|  | c.722G>A | p.E258K | missense | 0.06 | rs121912652/COSV52684909 | 0.42 |
| P30 | c.646G>A | p.V216M | missense | 0.03 | rs730882025/COSV52671096 | 0.48 |
| P31 | c.329G>C | p.R110P | missense | 0.63 | rs11540654/- | 0.88 |
|  | c.880G>T | p.E294* | nonsense | 0.03 | rs1057520607/- | 0.04 |
| P32 | c.839G>C | p.R280T | missense | 0.05 | rs121912660/COSV52666248 | 0.13 |
|  | c.743G>A | p.R248Q | missense | 0.03 | rs11540652/- | 0.08 |
| P33 | c.706T>C | p.Y236H | missense | 0.27 | rs587782289/COSV52783032 | 1 |
|  | c.903_904insC | p.G302fs | frameshift | 0.55 | -/- | 1 |
| P34 | c.742C>T | p.R248W | missense | 0.24 | rs121912651/COSV52662035 | 1 |

*For all mutations the CCF was calculated according Landau *et al*., 2013 (doi.org/10.1038/leu.2013.248).

Abbreviations: CCF: cancer clonal fraction, MM: multiple myeloma, UTR: untranslated region, VAF: variant allele frequency.

**Supplementary Table S6** Treatment regimens across multiple myeloma risk groups stratified by treatment lines.

| **Risk group** | **Treatment lines**  n (%) | | **ASCT**  **(n=43)**  n (%) | **PI based**  **(n=98)**  n (%) | **MoAb based**  **(n=41)**  n (%) | **IMID based**  **(n=11)**  n (%) | **Bispecific antibodies**  **(n=11)**  n (%) |
| --- | --- | --- | --- | --- | --- | --- | --- |
| **Multi-hit *TP53***  (n=24) | TN  1  2  ≤3 | 6 (25.0) | 2 (4.7) | 4 (4.1) | - | - | - |
|  |  | 6 (25.0) | 1 (2.3) | 2 (2.0) | 3 (7.3) | - | - |
|  |  | 5 (20.8) | - | 2 (2.0) | 2 (4.9) | 1 (9.1) | - |
|  |  | 7 (29.2) | - | - | 5 (12.2) | 1 (9.1) | 1 (9.1) |
| **HR**  (n=108) | TN | 75 (69.4) | 18 (41.9) | 51 (52.0) | 3 (7.3) | 2 (18.2) | 1 (9.1) |
|  | 1 | 18 (16.7) | 3 (7.0) | 3 (3.1) | 7 (17.1) | 1 (9.1) | 4 (36.4) |
|  | 2 | 5 (4.6) | - | 1 (1.0) | 3 (7.3) | 1 (9.1) | - |
|  | ≤3 | 10 (9.3) | - | 1 (1.0) | 6 (14.6) | 1 (9.1) | 2 (18.2) |
| **SR**  (n=72) | TN | 53 (73.6) | 16 (37.2) | 30 (30.6) | 2 (4.9) | 3 (27.3) | 2 (18.2) |
|  | 1 | 13 (18.1) | 2 (4.7) | 3 (3.1) | 6 (14.6) | 1 (9.1) | 1 (9.1) |
|  | 2 | 1 (1.4) | - | - | 1 (2.4) | - | - |
|  | ≤3 | 5 (6.9) | 1 (2.3) | 1 (1.0) | 3 (7.3) | - | - |

Abbreviations: ASCT: autologous stem cell transplantation; HR: high-risk; IMID: immunomodulatory drug; MoAb: monoclonal antibody; PI: proteasome inhibitor; SR: standard risk; TN: treatment-naïve.

**Supplementary Table S7** Distribution of treatment regimens across the risk groups.

| **Risk group** | **ASCT**  **(n=43)**  n (%) | **PI based**  **(n=98)**  n (%) | **MoAb based**  **(n=41)**  n (%) | **IMID based**  **(n=11)**  n (%) | **Bispecific antibodies**  **(n=11)**  n (%) |
| --- | --- | --- | --- | --- | --- |
| **Multi-hit *TP53***  (n=24) | 3 (7.0) | 8 (8.2) | 10 (24.4) | 2 (18.2) | 1 (9.1) |
| **HR**  (n=108) | 21 (48.8) | 56 (57.1) | 19 (46.3) | 5 (45.5) | 7 (63.6) |
| **SR**  (n=72) | 19 (44.2) | 34 (34.7) | 12 (29.3) | 4 (36.4.) | 3 (27.3) |

Abbreviations: ASCT: autologous stem cell transplantation; HR: high-risk; IMID: immunomodulatory drug; MoAb: monoclonal antibody; PI: proteasome inhibitor; SR: standard risk.

**Supplementary Table S8** The frequency of HR cytogenetic abnormalities in MM patients treated with ≤1 and ≥2 prior therapy lines.

| **HR cytogenetic abnormalities** | **≤1 line (n=171)**  n (%) | **≥2 lines (n=33)**  n (%) | *p** |
| --- | --- | --- | --- |
| accumulation of HR (≥ 2) cytogenetic abnormalities | 37 (21.6) | 8 (24.2) | 0.403 |
| del(1p) | 24 (14.0) | 2 (6.1) | 0.263 |
| t(4;14) | 17 (9.9) | 9 (27.3) | 0.018 |
| t(14;16) | 7 (4.1) | 1 (3.0) | 0.999 |
| 1q21 gain/amp | 73 (42.7) | 18 (54.5) | 0.241 |

**P*-values were estimated using Fisher’s exact test.

Abbreviations: HR: high risk, MM: multiple myeloma.

**Supplementary Table S9** The frequency of *TP53* aberrations, multi-hit *TP53* and mono-hit *TP53* aberrations in treatment-naïve and relapsed/refractory patients with MM.

| ***TP53* aberrations** | **Treatment naïve**  **(n=134)**  n (%) | **Relapsed/refractory (n=70)**  n (%) | *p** |
| --- | --- | --- | --- |
| *TP53* aberration | 22 (16.4) | 30 (42.9) | < 0.001 |
| Multi-hit *TP53* | 6 (4.5) | 18 (25.7) | < 0.001 |
| Mono-hit *TP53* | 16 (11.9) | 12 (17.1) | 0.391 |

*P-values were estimated using Fisher’s exact test.

Abbreviations: MM: multiple myeloma, ns: not significant.

**Supplementary Table S10** Overview of *TP53* abnormalities in paired bone marrow samples analysed at baseline and at relapse/progression in MM patients.

| **Patient** | **Del(17p) (VAF)** | | ***TP53*mut (VAF)** | | **Regiment** | **Therapy line** | **Time to progression (months)** |
| --- | --- | --- | --- | --- | --- | --- | --- |
|  | **Baseline** | **Relapse** | **Baseline** | **Relapse** |  |  |  |
| P1 | - | - | 0.33 | 0.64;  0.14 | ASCT | 1 | 38 |
| P2 | - | - | 0.02 | 0.23 | KRd | 2 | 37 |
| P3 | - | - | 0.04 | 0.08 | DRD | 3 | 25 |
| P4 | - | 0.80 | 0.04;  0.02 | 0.16; 0.40; 0.12* | VTD | 1 | 28 |
| P5 | - | 0.64 | 0.04 | 0.02 | DVD | 2 | 12 |
| P6 | - | 0.16 | 0.03 | 0.16; 0.08; 0.07;  0.19 | CTD | 3 | 15 |
| P7 | - | - | - | 0.26 | KRd | 2 | 32 |
| P8 | - | - | - | 0.03 | KRd | 3 | 30 |
| P9 | - | - | - | 0.02 | KD | 3 | 11 |
| P10 | - | - | - | 0.03 | RD | 3 | 22 |
| P11 | - | - | - | 0.23 | ASCT | 1 | 40 |
| P12 | - | - | - | 0.17;  0.06 | ASCT | 2 | 20 |
| P37 | 0.39 | 0.70 | - | - | ASCT | 1 | 24 |
| P38 | 0.91 | 0.85 | - | - | ASCT | 1 | 49 |
| P39 | 0.98 | 0.98 | - | - | ASCT | 1 | 19 |
| P40 | - | 0.50 | - | - | VTD | 1 | 52 |
| P41 | - | 0.60 | - | - | VRD | 1 | 6 |

*Different variants, for details see Table S5.

Abbreviations: ASCT: autologous stem cell transplantation; CTD: cyclophosphamide, thalidomide, dexamethasone; DRD: daratumumab, lenalidomide, dexamethasone; DVD: daratumumab, bortezomib, dexamethasone; KD: carfilzomib, dexamethasone; KRd: carfilzomib, lenalidomide, dexamethasone; MM: multiple myeloma; RD: lenalidomide, dexamethasone**; VAF**: variant allele frequency; VTD: bortezomib, thalidomide, dexamethasone; VRD: bortezomib, lenalidomide, dexamethasone.

**Supplementary Fig. S1** Flow diagram of analysed samples from patients with MM.

**
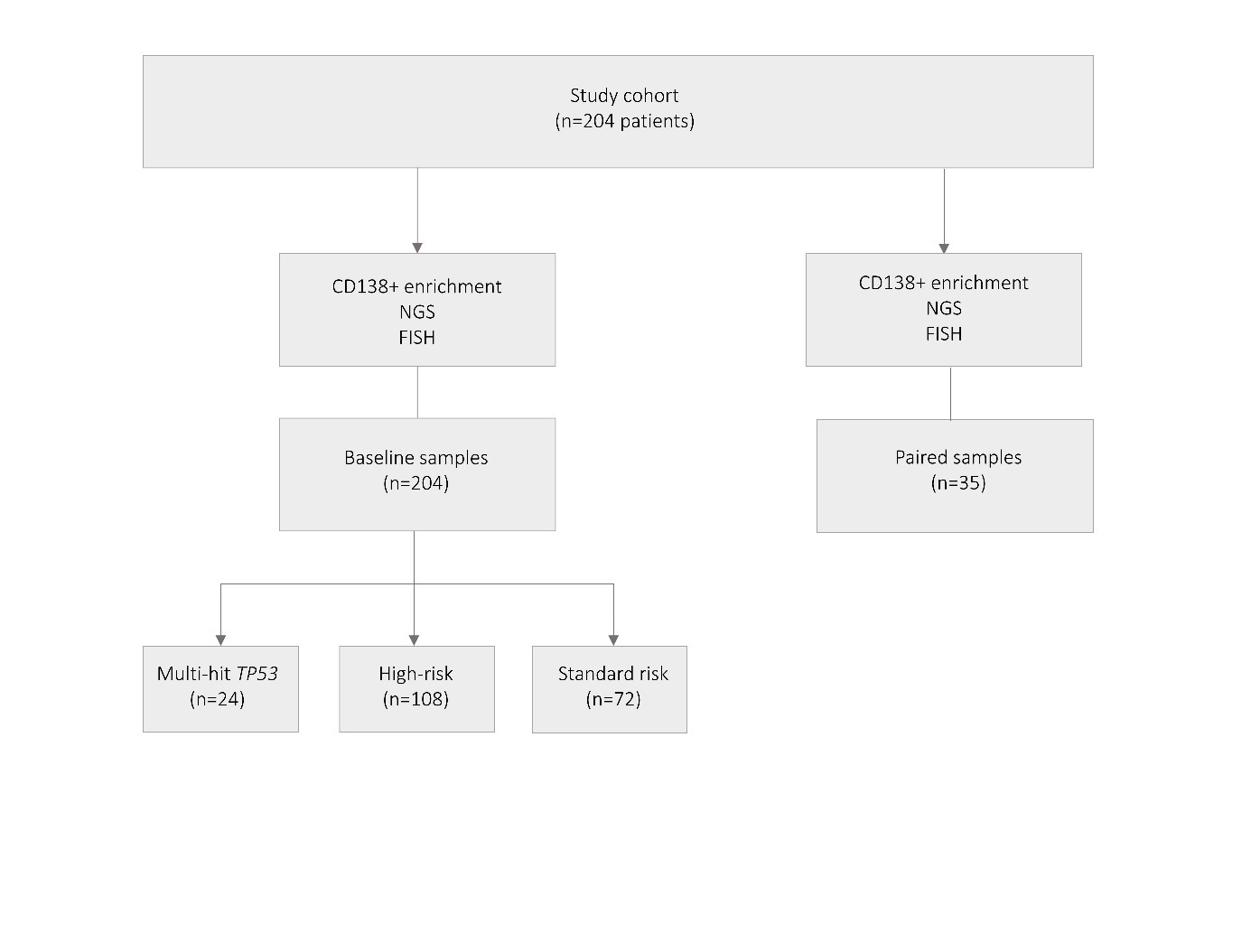
**

Abbreviations: FISH: fluorescence in situ hybridization; MM: multiple myeloma; NGS: next generation sequencing.

**Supplementary Fig. S2** Lollipop plot representing the location of detected *TP53*mut in all analysed samples.

NM_000546 was used as a reference transcript. The number in a circle shows how many times the variant occurred.


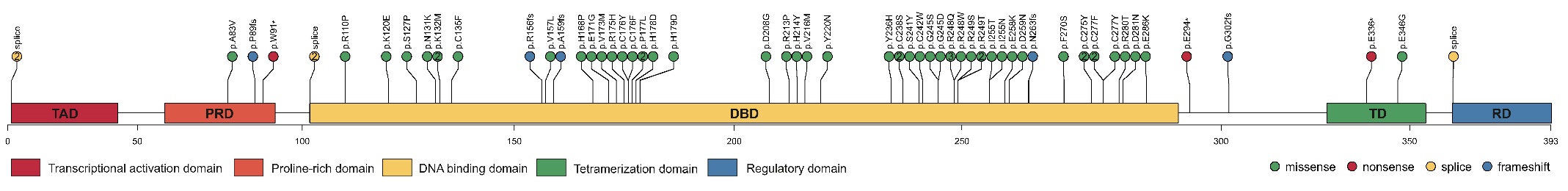


Abbreviations: DBD: DNA binding domain; PRD: Proline-rich domain; RD: Regulatory domain; TAD: Transcriptional activation domain; TD: Tetramerization domain.

**Supplementary Fig. S3** Multivariate Cox regression analysis of prognostic genetic abnormalities, treatment regimens and clinical parameters associated with overall survival in patients with MM.


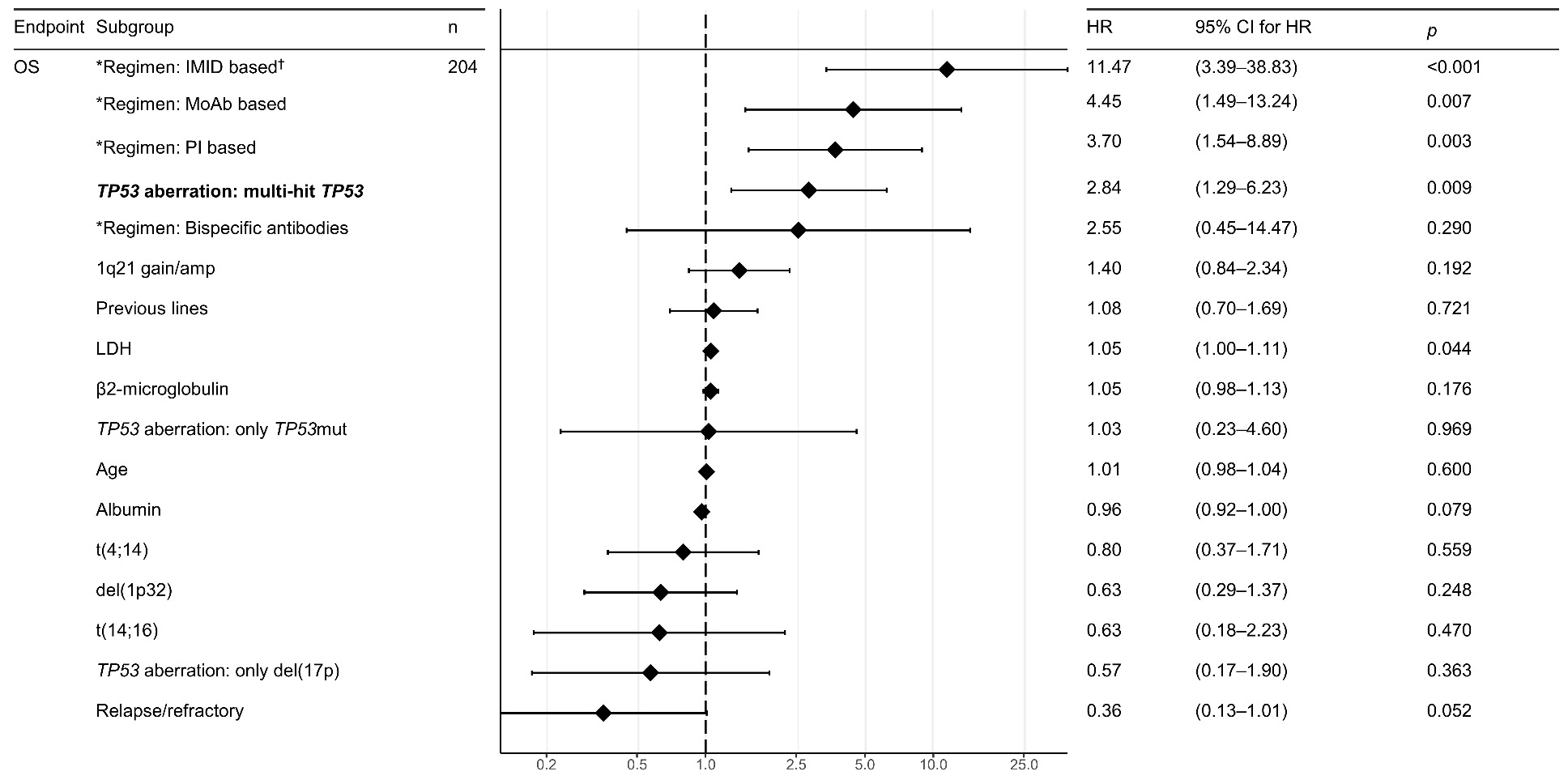


^*^ASCT was considered as a reference treatment group.

^†^Within the IMID-based group, all patients (n=11) were treated with a single agent only.

Abbreviations: ASCT: autologous stem cell transplantation; CI: confidence interval; HR: hazard-ratio; IMID: immunomodulatory drug; LDL: lactate dehydrogenase; MM: multiple myeloma; MoAb: monoclonal antibody; OS: overall survival; PI: proteasome inhibitor. **Supplementary Fig. S4** Progression-free survival of patients with multiple myeloma treated with different therapeutic regimens and stratified by risk into multi-hit *TP53*, HR, and SR groups.


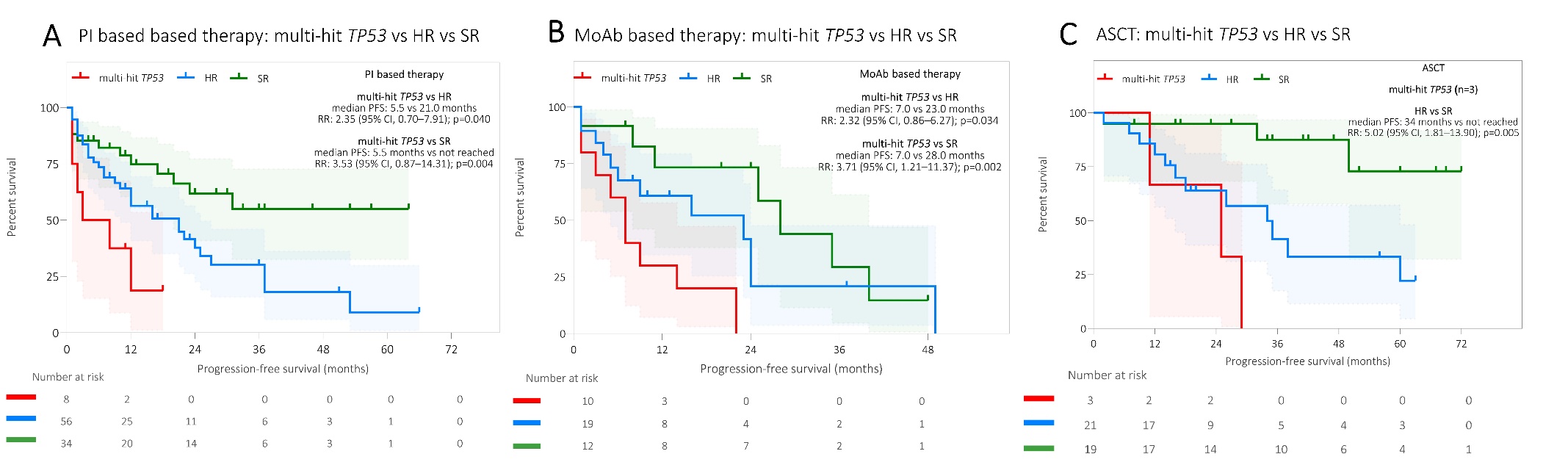


Abbreviations: ASCT: autologous stem cell transplantation; HR: high-risk; IMID: immunomodulatory drug; MoAb: monoclonal antibody; PI: proteasome inhibitor; SR: standard risk.

**Supplementary Fig. S5** Progression-free survival of treatment-naïve patients with MM. **A)** Patients with multi-hit *TP53* compared to HR and SR group. **B)** Patients with multi-hit *TP53* compared to ≥2 HR abnormalities, one HR abnormality and SR.


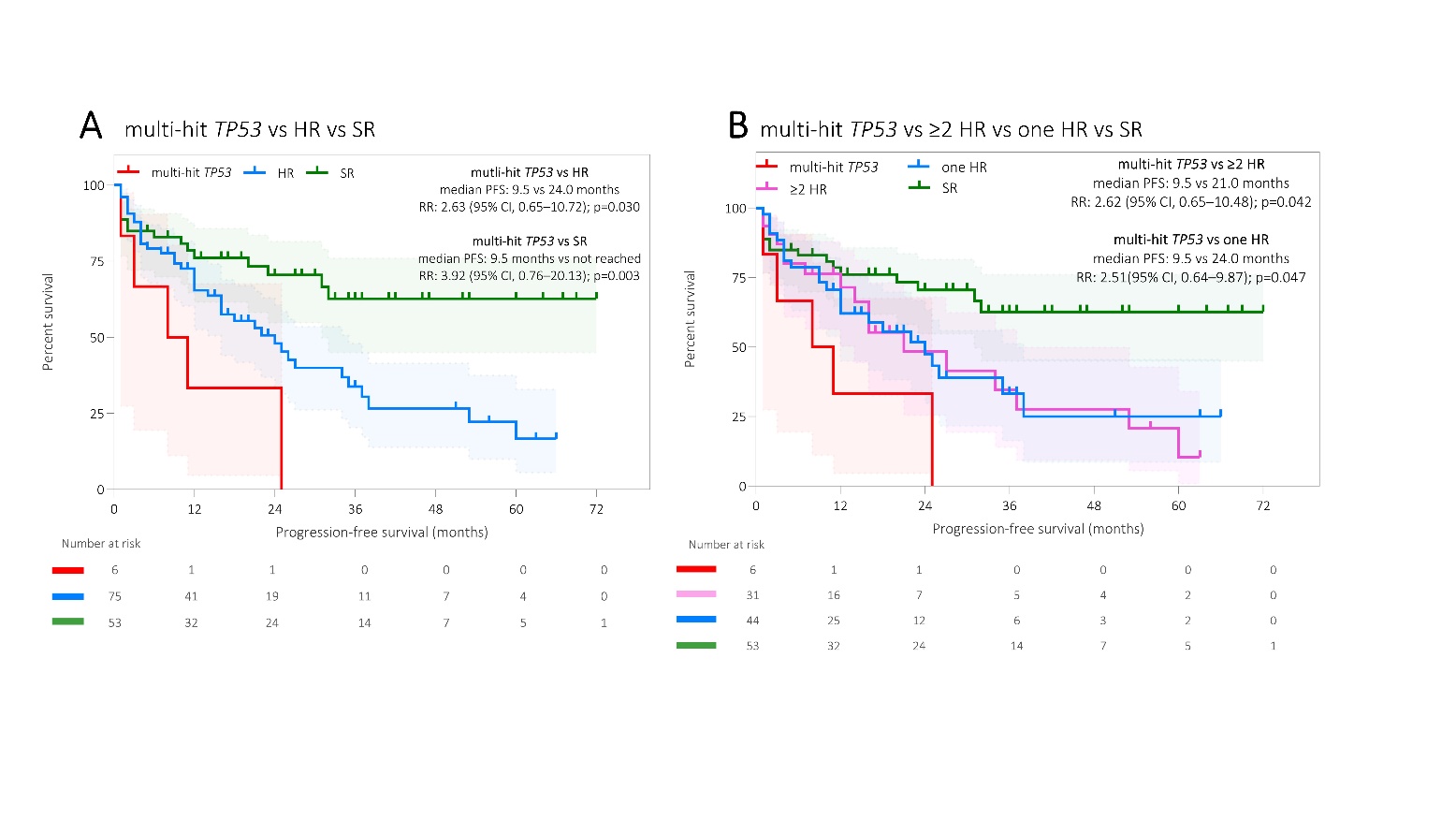


Abbreviations: HR: high-risk; MM: multiple myeloma; PFS: progression-free survival; SR: standard risk; TN: treatment-naïve.
